# ‘Put Your Money Where Your Mouth Is!’: Effects of Streaks on Confidence and Betting in a Binary Choice Task

**DOI:** 10.1002/bdm.1844

**Published:** 2014-10-28

**Authors:** Bettina Studer, Eve H Limbrick-Oldfield, Luke Clark

**Affiliations:** 1 Department of Psychology, University of CambridgeCambridge, UK; 2 Institute of Cognitive Neuroscience, University College LondonLondon, UK; 3 Centre for Gambling Research at UBC, Department of Psychology, University of British ColumbiaVancouver, Canada

**Keywords:** sequential biases, decision confidence, post-decision wagering, loss chasing

## Abstract

Human choice under uncertainty is influenced by erroneous beliefs about randomness. In simple binary choice tasks, such as red/black predictions in roulette, long outcome runs (e.g. red, red, red) typically increase the tendency to predict the other outcome (i.e. black), an effect labeled the “gambler's fallacy.” In these settings, participants may also attend to streaks in their predictive performance. Winning and losing streaks are thought to affect decision confidence, although prior work indicates conflicting directions. Over three laboratory experiments involving red/black predictions in a sequential roulette task, we sought to identify the effects of outcome runs and winning/losing streaks upon color predictions, decision confidence and betting behavior. Experiments 1 (*n* = 40) and 3 (*n* = 40) obtained trial-by-trial confidence ratings, with a win/no win payoff and a no loss/loss payoff, respectively. Experiment 2 (*n* = 39) obtained a trial-by-trial bet amount on an equivalent scale. In each experiment, the gambler's fallacy was observed on choice behavior after color runs and, in experiment 2, on betting behavior after color runs. Feedback streaks exerted no reliable influence on confidence ratings, in either payoff condition. Betting behavior, on the other hand, increased as a function of losing streaks. The increase in betting on losing streaks is interpreted as a manifestation of loss chasing; these data help clarify the psychological mechanisms underlying loss chasing and caution against the use of betting measures (“post-decision wagering”) as a straightforward index of decision confidence. © 2014 The Authors. *Journal of Behavioral Decision Making* published by John Wiley & Sons Ltd.

## Introduction

Gambling games typically involve a series of independent events such as successive draws in a lottery or the spins of a slot machine or roulette wheel. A wealth of psychological data over the past 50 years indicates that our predictions of these events show systematic deviations from randomness (Edwards, 1961; Jarvik, 1951; Oskarsson et al., 2009). Even within a task as simple as binary guessing, multiple influences of the recent event history can be observed, indicating a fundamental ignorance of the independence of turns. The most well-studied example is the “gambler's fallacy”: After a run of identical outcomes (e.g. red, red, red in roulette), people typically predict the other outcome (i.e. black) as due. The gambler's fallacy has been described in field data from gambling venues (Clotfelter & Cook, 1993; Croson & Sundali, 2005; Wagenaar, 1988) and investment decisions (refer to Oskarsson et al., 2009), as well as in laboratory studies (Ayton & Fischer, 2004; Barron & Leider, 2010; Boynton, 2003). The dominant account of this effect proposes that humans expect small sequences to be representative of the overall distribution from which the events are drawn. This “belief in the law of small numbers” is considered an example of the representativeness heuristic (Kahneman & Tversky, 1972). Related accounts discuss how the scarcity of truly independent events in the real world may lead to a folk intuition that gambling games involve sampling without replacement, in which the gambler's fallacy would be a reasonable assumption (Estes, 1964; Rabin, 2002) or that long runs may evoke a Gestalt need for closure (Roney & Trick, 2003).

In other settings, binary outcome predictions can display the opposite pattern, such that people predict that a series of identical events will continue rather than reverse. In the context of sports, if a player scores with three successive shots, spectators tend to expect the player to score with their next attempt; this was originally described in basketball and labeled the “hot hand” belief (Alter & Oppenheimer, 2006; Gilovich et al., 1985). Like the gambler's fallacy, the hot hand belief may be explained by reference to the representativeness heuristic: Observation of a streak may prompt us to reject the assumption that the sequence is random and conclude instead that the events must be dependent. While both explanations rely on representativeness, these phenomena may be distinguished by the mechanism that generates (or is thought to generate) the events. Croson and Sundali (2005) pointed out that the gambler's fallacy is observed following runs of outcomes generated by external devices such as roulette wheels, whereas the hot hand is classically observed where there is feedback upon human performance, like correct/incorrect guesses or gains and losses. Henceforth, we distinguish *outcome runs* and *feedback streaks* in this manner (refer also to Ayton & Fischer, 2004; Burns & Corpus, 2004).

The behavioral response to feedback streaks is less well-characterized than the gambler's fallacy following outcome runs. A seminal experiment by Ayton and Fischer (2004, experiment 1) measured subjective confidence following red/black color predictions in a laboratory roulette task. The gambler's fallacy was observed on color choice, such that the choice of either color decreased linearly as a function of the prior run length of that color. Confidence ratings, by contrast, were sensitive to feedback streaks and varied in line with the hot hand belief, increasing linearly on winning streaks and decreasing linearly on losing streaks. By contrast, studies of actual gambling behavior indicate a rather different response. Structured interviews and surveys with real-life gamblers describe a tendency to respond to losses by increasing one's bet; this is termed “loss chasing” (Dickerson et al., 1987; Lesieur, 1984; O'Connor & Dickerson, 2003). Using field data from a horse-racing track, McGlothlin (1956) observed that bet amounts tended to increase throughout the day, while the “track take” ensures that most punters gradually lose over time (refer also to Ali, 1977). More specifically, there was a negative correlation in McGlothin's data between the proportion of bettors who won on a given race and the amount bet per gambler on the subsequent race. In data from online poker, Smith et al. (2009) observed that experienced players responded to major losses (of over $1000 in a single hand) by becoming more aggressive on the next hand and betting on weaker hands to stay in the game. They responded to equivalently sized wins in the opposite manner.

These results present a contradiction if one accepts an emerging view that the amount bet on a decision provides a proxy for decision confidence. This index of “post-decision wagering” conveys several advantages over explicit reports of decision confidence, given its suitability for use in non-human species in behavioral neuroscience (Kepecs & Mainen, 2012) and the possibility for studying decision processes outside of awareness (Koch & Preuschoff, 2007; Persaud & McLeod, 2008; Persaud et al., 2007). Critically, this argument would predict a parallel effect of feedback streaks on betting behavior to that observed for subjective confidence. Thus, the findings from Ayton and Fischer (2004) would predict that bet sizes should *increase* following winning streaks and *decrease* following losing streaks, although the gambling literature reviewed in the preceding texts indicates the direct opposite pattern. The overarching aim of the three experiments described here was to characterize these differential sensitivities of decision confidence and betting behavior to feedback streaks, with a view to understanding the boundary conditions under which loss chasing can be observed in the laboratory.

Using a roulette task with binary red/black predictions, experiment 1 sought to establish the classic gambler's fallacy and replicate the effects of winning and losing streaks upon decision confidence described by Ayton and Fischer (2004). Experiment 2 tested the effects of winning and losing streaks upon betting behavior. Together, the results of the first two experiments indicated a differential effect of losing streaks upon betting behavior versus confidence ratings. Experiment 3 served as a control experiment to investigate whether this divergence could be explained by a difference in the payoff structure of the first two experiments. For experiment 1, our hypotheses were as follows: for color choice, we predicted that the probability of choosing the same color as the previous outcome would decrease as a function of the outcome run length, demonstrating the gambler's fallacy. We predicted that confidence ratings would be higher when choice was consistent with the gambler's fallacy. Moreover, we expected confidence ratings to increase as a function of the length of winning streaks and decrease as a function of the length of losing streaks, consistent with the hot hand belief.

## Experiment 1

### Methods

#### Participants

Forty healthy university students took part in a single testing session lasting approximately 45 min (19 males, 21 females, average age = 20 years, SD = ±1 year). The Problem Gambling Severity Index (Ferris & Wynne, 2001) was administered to all participants in order to screen for potential gambling problems. One participant was classified as a problem gambler (score = 8) and excluded from further analysis. The scores of all other volunteers were under 5, with the vast majority of participants (78%) scoring 0. Participants were reimbursed according to their final earnings on the task, with payments ranging between £3.80 and £5.80 (mean = £4.55). This and the two following studies were approved by the Psychology Research Ethics Committee of the University of Cambridge. In accordance with the Declaration of Helsinki, all participants gave written informed consent.

#### Task

**Figure 1:**
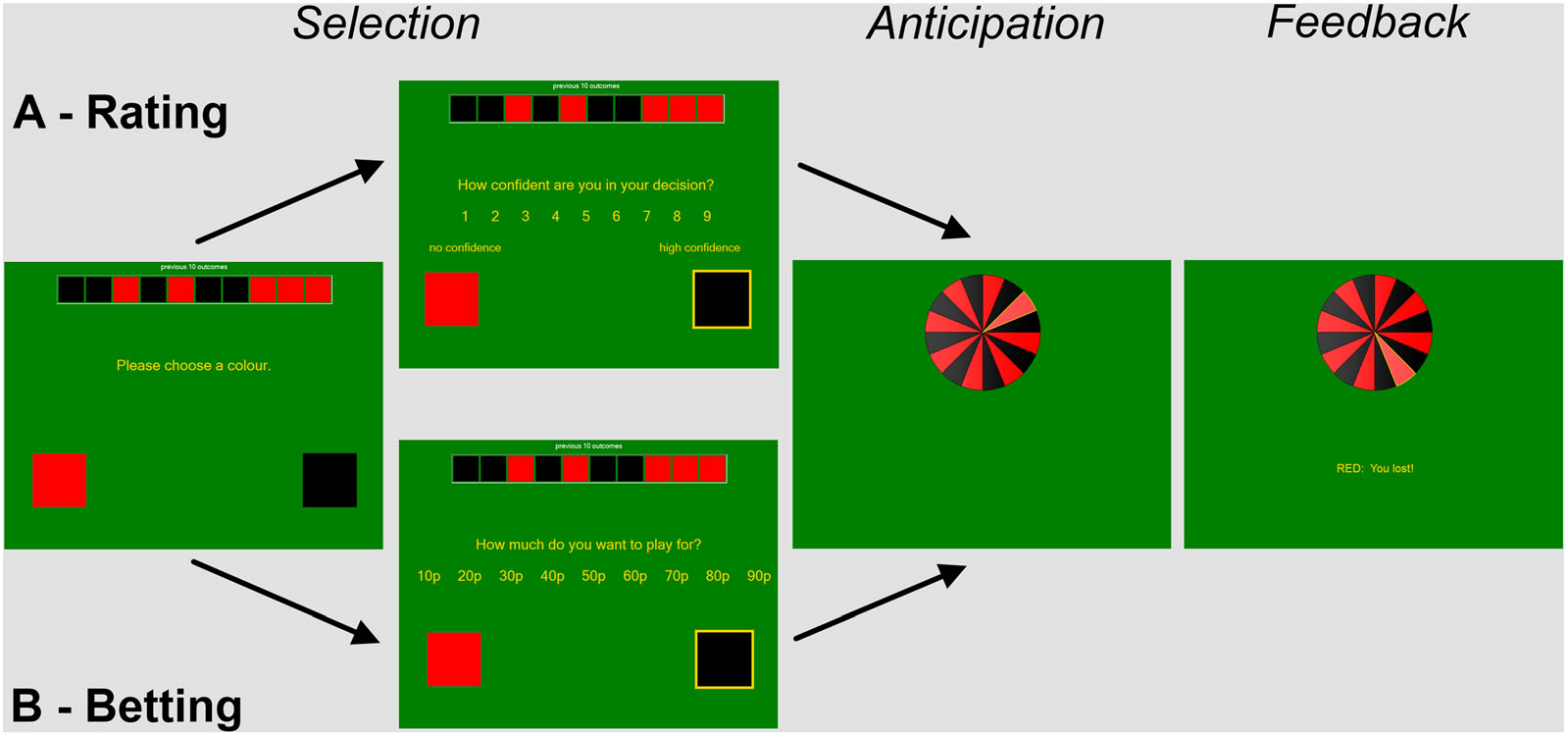
Experimental task each trial consisted of three phases: (1) selection, in which participants first made a color prediction and then rated their decision confidence (A, experiments 1 and 3) or placed a bet on that prediction (B, experiment 2), (2) anticipation, in which the wheel spun, (3) feedback, in which the decision outcome was presented

Participants completed three practice trials and 90 experimental trials on a computer-based roulette task modified from Ayton and Fischer (2004) and programmed in Visual Basics 2008 (Microsoft Corporation, USA). On each trial, participants were presented with a wheel consisting of 16 segments that were alternately black and red in color. Each trial consisted of a variable inter-trial interval (800–1200 ms), followed by a selection phase (no time limit), the wheel spin (1000 ms) and feedback window (1000 ms) (refer to Figure1). During the selection phase, participants made a red/black color prediction using two keys and then rated their confidence in that color prediction on a 9-point scale that ranged from “no confidence” to “high confidence.” During selection, a history panel presented the prior 10 red/black outcomes across the top of the display. The history panel is a common feature in casino roulette and was used in this context to minimize working memory demands, which may alter the processing of event sequences (Altmann & Burns, 2005). At the beginning of the task, the history panel was filled arbitrarily and progressively updated based on the delivered outcomes. Following the color prediction and confidence ratings, the roulette wheel spun for a short anticipation period and then stopped on one segment, initiating the feedback phase. Correct predictions were awarded 10 pence and accompanied by the message ‘You won’ and a cash-machine sound. Incorrect predictions generated the message ‘You lost’ with an unpleasant sound but no financial penalty.

The trial sequence was a fixed 90 trial pseudo-random sequence to ensure that participants experienced a sufficient number of longer color runs (refer to Table 1 for details). The sequence contained an equal number of black and red outcomes, with a probability of alternation of 0.48. To ensure that the pseudo-randomized sequence was not predictable, we compared participants' profits against chance performance using a one-sample t-test. Participants' performance did not differ significantly from chance (*t*_27_ = 0.85, *p* = .40).

**Table 1:** Frequencies of experienced run lengths and streak lengths [mean (SD)]

Run lengths		Winning streaks				Losing streaks			
Run length	All expts	Streak length	Expt 1	Expt 2	Expt 3	Streak length	Expt 1	Expt 2	Expt 3
1	44	1	23 (±2)	23 (±2)	23 (±2)	1	23 (±2)	23 (±2)	23 (±2)
2	24	2	11 (±2)	12 (±2)	11 (±2)	2	11 (±2)	11 (±2)	12 (±2)
3	9	3	6 (±2)	6 (±2)	5 (±2)	3	5 (±1)	5 (±2)	5 (±2)
4	7	4	3 (±1)	3 (±1)	3 (±2)	4	3 (±1)	3 (±1)	2 (±1)
5	5	>4	2 (±2)	2 (±2)	2 (±2)	>4	3 (±2)	2 (±2)	1 (±2)

Nb. run length was experimentally controlled and thus did not vary between experiments or participants. Feedback was not under experimental control, and therefore, the average frequency for each level of winning and losing streak length is presented.

#### Data analysis

For each trial, the color prediction and confidence rating were recorded. Confidence ratings were then classified as higher (1) or lower (0) than the individual participant's average. Trial-by-trial data was analyzed using logistic regression in R (R Core Team, Vienna, Austria). Three primary logistic regression models were created. Model 1 tested for the gambler's fallacy, by assessing run length (i.e. number of consecutive identical color outcomes preceding the trial: 1–5) as a potential predictor of color choice (same (1) or different (0) from previous outcome). Model 2 tested whether confidence ratings (1, 0) were also sensitive to the gambler's fallacy, by including run length (1–5), color choice (1, 0), and the interaction of these two factors as predictors. Hence, this model tested our second hypothesis that the gambler's fallacy would increase confidence when predicting against the previous color following long outcome runs, reflected statistically in run length × color choice interaction term. Model 3 assessed the effects of winning and losing streaks upon confidence ratings, by including previous feedback (win (1), loss (0)), streak length (1–4), and the interaction of these two factors as predictors. Because correct or incorrect guesses were not experimentally controlled, streak lengths of 5 or more were removed from these analyses, resulting in the exclusion of 5.6% of all trials. Because we hypothesized that winning and losing streaks would affect confidence in opposite ways, the primary effect of interest in this analysis is the streak length × previous feedback interaction.

For all models, the inputted data consisted of a separate data point for every trial and participant, and all models were conducted in two hierarchical steps (Field et al., 2012). First, participant was included as a categorical predictor, as we considered the general tendency to reselect the previous outcome to be a trait characteristic and thus best represented on a participant-by-participant basis. This model provides a beta value for each participant (not reported), which was arbitrarily compared with the first participant entered.^1977^ At the second step, the experimental variables of interest were added to this model. Run and streak lengths were entered as linear variables and tested for the linearity assumption of the logit. Multicollinearity of predictors was tested by calculating the variance inflation factor and tolerance. The odds ratio (OR) and the 95% confidence interval (95% CI) for this estimate were calculated for each predictor. Model fit was assessed with pseudo *R*^2^ using the Cox–Snell statistic, and the improvement to the model was quantified by comparing the residual deviance (with significant predictors included in the model) to the deviance with only the constant and participant included (henceforth called the null model), using a chi-squared test. Leverage and standardized residuals were calculated on a case wise basis, to identify cases that had undue influence on the model or where the model fit was poor. Unless stated otherwise, all assumptions of the model were met, and analysis of the residuals indicated the model was reliable and was not unduly influenced by any single cases. Only significant predictors were included in the final logistic regression models. The statistics for non-significant predictors are reported prior to their removal from the model. Given that we were primarily interested in assessing whether variation in color choice and confidence ratings was dependent upon color runs and winning/losing streaks, participants who consistently chose the same color and/or gave the same confidence rating (across 89 or 90 trials) were excluded from statistical analysis. In experiment 1, 12 participants (30%) were excluded on this basis.

### Results

#### Effects of outcome runs

**Figure 2:**
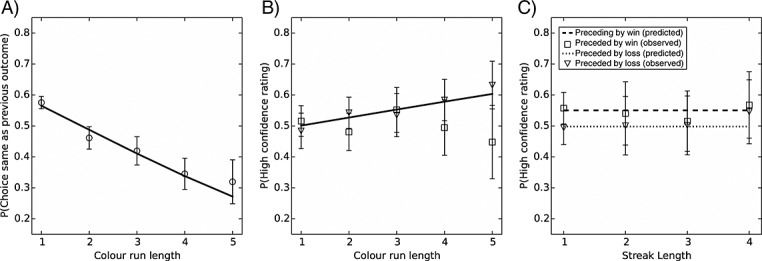
Results of experiment 1. (A) Effects of run length upon choice behavior. Graph shows the predicted (line) and observed (circles) probabilities of choosing the same color as won on the preceding trial for each run length. (B) Influence of run length upon confidence ratings. The predicted probability of providing a high confidence rating (line) increased with run length. Observed group averages are shown separately for trials in which participants predicted the same (squares) versus the different (triangles) color as won on the last trial. Note that no significant effects of color prediction or color prediction × run length were found. (C) Effect of feedback streaks upon decision confidence. The graph depicts the predicted probabilities of providing a high rating as a function of the length of preceding winning (dashed line) and losing (dotted line) streaks. The length of winning and losing streaks did not significantly predict confidence ratings. Observed group averages of confidence ratings following winning (squares) and losing (triangles) streaks are also displayed. For all graphs, error bars represent SEM of the observed group data

Color choice was significantly predicted by run length, *β*(*SE*) = −0.33 (0.04), *p* < .001. As run length increased, the predicted probability of choosing the same color as the last outcome decreased, OR = 0.72, 95% CI [0.66, 0.77] (refer to Figure2A). The pseudo *R^2^* was 09 (Cox–Snell), and the addition of the run length predictor significantly improved model fit over the null model, ***χ***^2^(1) = 77.6, *p* < .001.

Confidence ratings were significantly predicted by run length, *β*(*SE*) = 0.14 (0.04), *p* < .001, such that longer runs were associated with increased confidence, OR = 1.15, 95% CI [1.06, 1.25] (refer to Figure2B). The pseudo *R*^2^ was 27 (Cox–Snell). Color choice was not a significant predictor, *β*(*SE*) = 0.35 (0.19), *p* = .07, OR = 1.41, 95% CI [0.97, 2.05], and the run length × color choice interaction term was not significant, *β*(*SE*) = −0.16 (0.09), *p* = 0.06, OR = 0.85, 95% CI [0.72, 1.00]. Addition of the run length predictor significantly improved model fit over the null model, *χ*^2^(1) = 11.54, *p* < .001.

#### Effects of feedback streaks

Confidence ratings did not vary reliably as a function of streak length, *β*(*SE*) = 0.05 (0.04), *p* = .47, OR = 1.06, 95% CI [0.91, 1.22]. There was a significant effect of previous feedback, *β*(*SE*) = 0.28 (0.10), *p* < 0.01, OR = 1.33, 95% CI [1.10, 1.60], with higher ratings on trials following wins than on trials following losses, but previous feedback did not interact with streak length, *β*(*SE*) = −0.08 (0.14), *p* = .43, OR = 0.92, 95% CI [0.75, 1.13] (refer to Figure2C). The pseudo *R*^2^ was 27 (Cox–Snell), and addition of the previous feedback factor significantly improved model fit over the null model, *χ*^2^(1) = 8.71, *p* < .01.

### Discussion

In this first experiment, we observed a reliable effect of color runs on participants' predictions, such that choice of the previous outcome decreased as a function of run length. This is a classical gambler's fallacy effect, as seen in numerous prior studies (Ayton & Fischer, 2004; Barron & Leider, 2010; Jarvik, 1951). The incremental change in choice behavior as a function of run length was approximately linear (refer to Figure2A), similar to previous findings by Ayton and Fischer (2004) and Barron and Leider (2010). Subjective confidence ratings increased at longer run lengths. From inspection of Figure2B, it appears that the elevated confidence following longer runs was most evident when participants choose against the last outcome, which would constitute a further instantiation of the gambler's fallacy. We note however that the interaction term (run length × color prediction) only approached statistical significance (*p* = .07).

Confidence ratings were generally higher following wins compared with losses, but this effect did not vary as a function of the length of prior winning or losing streak. As such, these data do not support our hot hand hypothesis and fail to replicate the earlier results by Ayton and Fischer (2004); some methodological factors to explain this divergence are considered in the General discussion section. Experiment 2 moved away from confidence ratings to test whether feedback streaks systematically influence betting behavior. In this experiment, participants were asked to place a bet upon their color predictions. Thus, we effectively asked our participants to “put your money where your mouth is,” reasoning that the direct financial consequences of a bet may enhance its sensitivity to streak effects, relative to the subjective and inconsequential ratings employed in experiment 1. We predicted that color predictions would follow the gambler's fallacy, as in experiment 1, and that bet amounts would be greatest when color choice was consistent with the gambler's fallacy. We further predicted that winning and losing streaks would systematically influence betting behavior. This conflicting nature of the existing literature did not allow a clear directional prediction for this hypothesis: Past studies of gambling behavior indicate loss chasing, with increased betting following losing streaks and no strong prediction for winning streaks. However, assuming equivalence of betting behavior and subjective confidence, the Ayton and Fischer (2004) study predicts that winning streaks would increase the amount bet and losing streaks would decrease the amount bet.

## Experiment 2

### Methods

#### Participants

Thirty-nine healthy university students (18 males, 21 females, average age = 20 years, SD = ±1 year) who had not participated in experiment 1 were recruited. No participants were classified as problem gamblers.

#### Task

A single modification was made to the task in experiment 1: After the color prediction, participants were now asked to place a bet on their color choice, in place of the subjective confidence rating. The bet response was presented on an equivalent 9-point scale ranging from 10p to 90p, in 10p increments (refer to Figure1). If the color prediction was correct, the amount bet was added to their points score, and if their prediction was wrong, the bet was deducted, such that higher bets were associated with greater outcome variance, and the expected value of any bet was zero. To accommodate the possibility of losing money in the task, participants were endowed initially with £5, and their final score was paid on completion (mean = £5.15, range £0 to £13.30).

#### Data analysis

Data analysis followed experiment 1, with trial-by-trial bet size, coded as higher (1) or lower (0) than that participant's mean bet, replacing the confidence rating. Three logistic regression models were run, as described in experiment 1. Eleven participants (28%) were excluded who consistently chose the same color and/or placed the same bet, and trials containing a streak length of 5 or more (4.4% of all trials) were excluded from the regression models.

### Results

#### Effects of outcome runs

**Figure 3:**
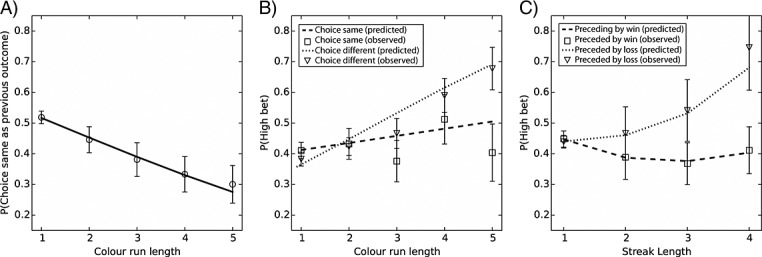
Results of experiment 2. (A) Effects of run length upon choice behavior. Graph shows the predicted (line) and observed (circles) probabilities of choosing the same color as won on the preceding trial for each run length. (B) Influence of Gambler's fallacy upon betting behavior. The graph depicts the interaction effect between run length and color prediction: The predicted probabilities of betting high increase more steeply with run length when participants predicted that the run would be terminated (dashed line) compared with when they predicted that the run would continue (dotted line). Group means of observed probabilities are also shown for choice same as (squares) or different from (triangles) last outcome. (C) Differential effect of losing versus winning streaks upon betting behavior. The graph depicts the probability of betting higher than average as a function of streak length. For losing streaks, the predicted (dotted line) and observed (triangles) probabilities of betting high increased as streaks grew longer. In contrast, betting behavior was not significantly influenced by the length of winning streaks (predicted probabilities—dashed line, observed probabilities—squares). For all graphs, error bars represent SEM of the observed mean data

Color choice was significantly predicted by run length, *β*(*SE*) = −0.28 (0.04), *p* < .001. The predicted probability of choosing the same color as the previous outcome decreased as a function of run length, OR = 0.75, 95% CI [0.70, 0.81] (refer to Figure3A). The pseudo *R^2^* was 11 (Cox–Snell), and the addition of the run length predictor significantly improved model fit over the null model, ***χ***^2^(1) = 57.64, *p* < .001.

Betting behavior was significantly predicted by run length, *β*(*SE*) = 0.35 (0.05), *p* < .001, OR = 1.41, 95% CI [1.29, 1.55], such that longer runs were associated with a greater likelihood of a high bet, and by color choice, *β*(*SE*) = 0.44 (0.17), *p* < .01, OR = 1.56, 95% CI [1.13, 2.15], with higher bets when choosing against the previous outcome. There was also a significant interaction of run length and color choice, *β*(*SE*) = −0.25 (0.08), *p* = .01, OR = 0.78, 95% CI [0.67, 0.90], such that the likelihood of a high bet was greatest when choosing against the previous outcome following a long outcome run (refer to Figure3B). The pseudo *R*^2^ was 05 (Cox–Snell), and inclusion of the three predictors significantly improved model fit over the null model, ***χ***^2^(3) = 61.71, *p* < .001.

#### Effects of feedback streaks

Evaluation of the logistic regression model 3 showed that the predictor streak length violated the linearity assumption. Therefore, streak length was treated as a categorical predictor in this analysis, and each level of streak length (2–4) was compared with a streak length of one. Betting behavior was differentially affected by winning versus losing streaks, as indicated by a streak length × previous feedback interaction (refer to Figure3C). This interaction term reach statistical significance at a streak length of three, *β*(*SE*) = −0.68 (0.26), *p* < .01, OR = 0.51, 95% CI [0.31, 0.84], and four, *β*(*SE*) = −1.21 (0.37), *p* < .001, OR = 0.30, 95% CI [0.14, 0.61], but not at a streak length of two, *β*(*SE*) = −1.21 (0.37), *p* = .08, OR = 0.72, 95% CI [0.48, 1.04]. Streak length also emerged as a significant predictor (streak length of three, *β*(*SE*) = 0.38 (0.11), *p* < .05, OR = 1.46, 95% CI [1.01,2.10], of four, *β*(*SE*) = 0.03 (0.29), *p* < .001, OR = 2.78, 95% CI [1.61, 4.98], and of two, *β*(*SE*) = 0.08 (0.14), *p* = .54, OR = 1.09, 95% CI [0.83, 1.42]). Addition of the predictors streak length and streak length × previous feedback significantly improved model fit over the null model, *χ*^2^(7) = 27.94, *p* < .001. Pseudo *R*^2^ was 03 (Cox–Snell). Previous feedback (in isolation) was not a significant predictor of betting behavior, *β*(*SE*) = 1.03 (0.29), *p* = .81, OR = 1.03, 95% CI [0.83, 1.28].

To decompose the streak length × previous feedback interaction term, separate regression models were run for winning and losing streaks. Betting behavior was significantly predicted by the length of losing streaks, *β*(*SE*) = 0.28 (0.07), *p* < .001, such that longer losing streaks were associated with an increase in the predicted probability of placing a high bet, OR = 1.32, 95% CI [1.15, 1.51]. In contrast, betting did not vary significantly as a function of the length of winning streaks, *β*(*SE*) = −0.12 (0.07), *p* = .07, OR = 0.89, 95% CI [0.78, 1.01].

### Discussion

The gambler's fallacy from experiment 1 was confirmed in participant's color choices. Betting behavior was also predicted by a regressor representing the run length by color choice interaction, such that the predicted probability of placing a high bet was greatest after longer outcome runs when the participant committed the gambler's fallacy. This result demonstrates the sensitivity of the bet measure and also provides a useful indication that the gambler's fallacy extends to risky decisions that carry direct financial consequences for the participant.

Notably, experiment 2 found that betting behavior was significantly predicted by the length of losing streaks. In line with the demonstrations of loss chasing from the gambling literature, the probability of placing a high bet *increased* as losing streaks grew longer. This systematic effect of streak length extends prior laboratory work in which risk taking increases following a single loss compared with a single gain (Demaree et al., 2012; Gehring & Willoughby, 2002; Leopard, 1978). The change in betting behavior following losses was not observed following wins. This was reflected in a significant previous feedback × streak length interaction, and there was no reliable change in the amount bet as function of the length of winning streaks.

Taken together, the results of experiments 1 and 2 imply differential sensitivities of betting behavior versus subjective confidence ratings to losing streaks. However, there was also a pertinent methodological difference in the payout structure of the tasks in these two experiments: The betting measure in experiment 2 yielded both financial gains and losses, whereas in experiment 1, correct color predictions resulted in a monetary win while incorrect guesses resulted in no financial penalty. The absence of absolute losses in experiment 1 may have selectively reduced the sensitivity of that design to the effect of losing streaks. Experiment 3 tested this alternative explanation by obtaining subjective confidence ratings with an inverted payoff structure, such that incorrect guesses were financially penalized (−10 pence) and correct guesses avoided any loss. As for experiment 1, we expected to observe a gambler's fallacy effect on color predictions, increased confidence when choice was consistent with the gambler's fallacy, and based on Ayton and Fischer (2004), we predicted that confidence ratings would increase as a function of the length of winning streaks and decrease as a function of the length of losing streaks.

## Experiment 3

### Methods

#### Participants

Forty undergraduate students took part in the study (22 males, 18 females, age M = 20 years, SD = 1.0), who had not participated in the prior experiments. No participants were classified as problem gamblers.

#### Task

Participants completed an identical task to experiment 1 (refer to Figure1), with confidence ratings taken on a 9-point Likert scale from “no confidence” to “high confidence.” The only modification was to the payout structure, such that incorrect guesses lead to a 10p deduction and correct guesses carried no financial outcome. To obtain comparable overall profits to experiment 1, participants received an initial endowment of £9, and their final earnings ranged between £3.30 and £5.90 (mean = £4.40).

#### Data analysis

Data analysis followed experiment 1. Nine participants (22%) were excluded who consistently chose the same color and/or gave the same confidence rating, and trials containing a streak length of 5 or more (5.8% of all trials) were excluded from the regression models.

### Results

#### Effects of outcome runs

**Figure 4:**
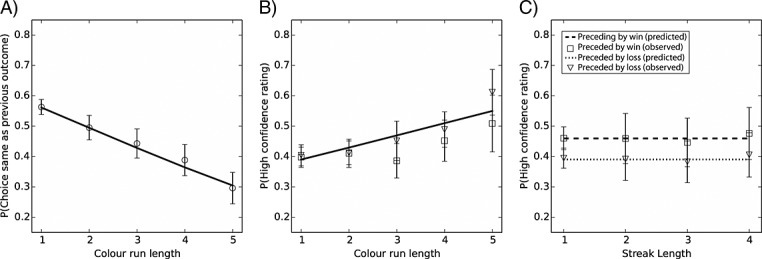
Results of experiment 3. (A) Effects of run length upon choice behavior. Graph displays the predicted (line) and observed (circles) probabilities of choosing the same color as won on the preceding trial for each run length. (B) Influence of run length upon confidence ratings. The predicted probability of providing a high confidence rating (line) increased with run length. Observed group data are shown separately for trials in which participants predicted the same (squares) versus the different (triangles) color as won on the last trial. Note that no significant effects of color prediction or color prediction × run length were found. (C) Effect of feedback streaks upon decision confidence. The graph depicts the predicted probabilities of providing a high rating as a function of the length of preceding winning (dashed line) and losing (dotted line) streaks. No systematic effects of streak length were found. Observed group averages of confidence ratings following winning (squares) and losing (triangles) streaks are also displayed. For all graphs, error bars represent SEM of the observed group data

Color choice was significantly predicted by run length, *β*(*SE*) = −0.30 (0.04), *p* < .001, such that longer runs were associated with a lower predicted probability of choosing the same color as the previous outcome, OR = 0.74, 95% CI [0.69, 0.80] (refer to Figure4A). The pseudo *R*^2^ was 11 (Cox–Snell), and the addition of the run length predictor significantly improved the model fit over the null model, ***χ***^2^(1) = 66.37, *p* < .001.

Confidence ratings were also significantly predicted by run length, *β*(*SE*) = 0.18 (0.04), *p* < .001, such that longer runs were associated with increased confidence, OR = 1.20, 95% CI [1.12, 1.29] (refer to Figure4B). The pseudo *R*^2^ was 11 (Cox–Snell). Confidence was not significantly predicted by color choice, *β*(*SE*) = 0.13 (0.16), *p* = .43, OR = 1.14, 95% CI [0.83, 1.57], or by the run length color choice interaction term, *β*(*SE*) = −0.12 (0.07), *p* = .10, OR = 0.89, 95% CI [0.76, 1.02]. The inclusion of the run length predictor significantly improved model fit over the null model, *χ*^2^(1) = 24.70, *p* < .001.

#### Effects of feedback streaks

As in experiment 1, confidence ratings did not vary reliably as a function of streak length, *β*(*SE*) = −0.01 (0.07), *p* = .85, OR = 0.99, 95% CI [0.87, 1.12]. Previous feedback was a significant predictor of confidence ratings, *β*(*SE*) = 0.32 (0.08), *p* < 0.01, OR = 1.37, 95% CI [1.11, 1.62], with higher ratings on trials following wins compared with trials following losses, but previous feedback did not interact with streak length, *β*(*SE*) = −0.01 (0.09), *p* = .93, OR = 0.99, 95% CI [0.83, 1.19] (refer to Figure4C). The pseudo *R*^2^ for the model was 11 (Cox–Snell), and addition of the factor previous feedback significantly improved model fit over the null model, ***χ***^2^(1) = 14.07, *p* < .001.

#### Direct comparison of betting versus confidence ratings following losing streaks

Experiments 1 to 3 indicate qualitatively different effects of losing streaks on the measure of betting as opposed to subjective confidence. To directly compare the effects across the three experiments, we ran a logistic regression model on the combined datasets.^2006^ “Measure” was entered as a binary predictor contrasting experiments 1 and 3 (confidence) against experiment 2 (betting). Losing streak length and the measure × losing streak length interaction term were also entered. The differential effect was supported by a significant losing streak length by measure interaction term, *β*(*SE*) = 0.28 (0.07), *p* < .001, OR = 1.32, 95% CI [1.15, 1.51], with losing streaks leading to an increase in bets but not in confidence ratings. The pseudo *R*^2^ was 19 (Cox–Snell), and the inclusion of the predictors losing streak length and measure was associated with significantly improved model fit over the null model, ***χ***^2^(1) = 15.9, *p* < .001.

### Discussion

The results of experiment 3 were qualitatively identical to those of experiment 1. There was a reliable gambler's fallacy on choice behavior, such that participants were less likely to choose either color following a longer run of that color. As in experiment 1, participants were more confident on decisions following win outcomes compared with loss outcomes, but confidence did not vary as a function of the *length* of losing or winning streaks. Thus, even with the reversal of the payoff structure such that incorrect guesses resulted in financial loss, there was no reliable fluctuation in confidence ratings in line with a hot hand effect, and confidence ratings did not follow the pattern of betting following losing streaks observed in experiment 2. Evidently, the experience of absolute losses is not the driving factor behind the differential findings for confidence ratings and betting.

We note that experiment 2 used a mixed payout structure with both gains and losses, whereas experiments 1 and 3 used a gain-only or loss-only payout structure, respectively. Previous research has shown that the payout domain can affect risk preference (Kuhberger et al., 1999), but it is difficult to conceive why the mixed structure would generate an effect that was restricted to losing streaks. We note that participants are typically risk averse for mixed gambles of equal probability (Schoemaker, 1990; Tversky & Kahneman, 1992) or at least risk neutral (Yechiam & Ert, 2011), whereas increased betting following losing streaks is evidently risk seeking.

In summary, the results of experiment 3 strengthen the assertion that the increase in betting in experiment 2 reflects the particular sensitivity of this measure to losing streaks that is distinct from subjective confidence. These differential sensitivities were further substantiated by a significant interaction of losing streak length and the measure taken, in a combined analysis across the three experiments.

### General discussion

The current study assessed the effects of outcome runs and feedback streaks on gambling behavior in a simulated roulette game with binary outcomes. Choice behavior in each of the three experiments adhered to the classic gambler's fallacy, whereby participants chose a given outcome less following a longer run of that outcome. The most striking effect of feedback streaks was seen in experiment 2, where the likelihood of placing a high bet increased on losing streaks. This pattern is in line with studies of naturalistic gambling, which have documented increased risk taking following losses (McGlothlin, 1956; O'Connor & Dickerson, 2003; Smith et al., 2009). Our data provide evidence that this effect is cumulative, as a function of the length of losing streaks (refer also to Ball, 2012; Leopard, 1978). The effect contrasts with the findings from experiments 1 and 3, in which confidence ratings were sensitive to the immediately previous outcome (greater following wins) but showed no significant fluctuation with the length of either kind of feedback streak. An increase in betting after losses is also the opposite pattern to prior demonstrations of a hot hand effect on confidence judgments (Ayton & Fischer, 2004; Sundali & Croson, 2006).

Several aspects of these data point to a parsimonious explanation in terms of loss chasing. First, an *increase* in betting serves directly to recover the recent losses. Second, this pattern was not observed for subjective confidence, plausibly because placing a high confidence rating carries no financial consequences. Third, the change in betting was observed for losing streaks and was not a statistically reliable effect for winning streaks. Loss chasing is regarded as the cardinal sign of the problem gambler (O'Connor & Dickerson, 2003; Toce-Gerstein et al., 2003), and it is often described as a desperate bid to recoup mounting debts (Lesieur, 1984; Walker, 1992). Our data help to arbitrate between two mechanistic accounts of loss chasing. One draws upon the same notion of negative recency as the gambler's fallacy: As the losing streak develops, the player believes that the streak must break and that a win is due. In a seminal book, Lesieur (1984 p. 52) writes that ‘if losses persisted, the gamblers could then latch onto “losing streak” explanations’; Sundali and Croson (2006) later referred to this as the “stock of luck” belief. Drawing upon brain imaging data, Campbell-Meiklejohn et al. (2008) argued that ‘loss chasing is mediated by activity in neural systems that represent an expectation of positive outcomes’ (pg 296). The current findings are difficult to reconcile with this account. If the increase in betting in experiment 2 reflected a heightened expectancy of winning, we would expect to see a similar increase in decision confidence following longer losing streaks in experiments 1 and 3. This was not observed. The “stock of luck” mechanism would also predict a mirrored effect after winning, that players should decrease their betting behavior because their good luck must run out; this was not a robust effect in the present data.

The alternative account—and one that is supported by the current data—draws upon prospect theory, that losing streaks enhance the subjective value of the potential win (Coval & Shumway, 2005; Kahneman & Tversky, 1979; Smith et al., 2009).^2005^ This is explained by the convex nature of the value function in the loss domain. As the losing streak moves the player progressively further from the reference point of 0, the value of a significant win increasingly exceeds the negative value of a further loss. This formulation is extended in recent work by Imas (2014), who finds that the increase in risk taking following losses is eradicated by “realizing” those losses, i.e. the actual transfer of money, as opposed to the “paper losses” that are inherent to a running tally. The realization of losses may prompt participants to close their mental account and re-center them to the (prospect theory) reference point. Within this account, the selectivity of our effect to losing streaks is explained by the standard asymmetry that ‘losses loom larger than gains’ (Kahneman & Tversky, 1979). We recognize that there is some overlap between this explanation of loss chasing and other effects in the decision-making literature. Financial loss chasing may be considered a specific instance of the “sunk cost effect” (Arkes & Blumer, 1985), describing the tendency to continue a no-longer profitable project that one has invested in. The “break even” hypothesis (Thaler & Johnson, 1990) predicts increased betting after losses in a specific effort to “balance the books”, i.e. to return to the original reference point (refer also to Heath, 1995). In our task, we did not display a trial-by-trial score in order that immediate streak history would not be confounded by the cumulative earnings (refer to Elliott et al., 2000). As such, we suspect that our participants were not formally aware of “breaking even”, although Heath (1995) notes that “escalating commitment” (akin to loss chasing) tends to be greatest under conditions where investments are hard to track, which would apply to our task.

Our findings speak to a larger literature on the assessment of decision confidence, which posits that betting behavior (post-decision wagering) can serve as a proxy measure of subjective confidence, for decisions that are outside of awareness (Persaud et al., 2007) or in non-human species that are unable to provide explicit judgments (Kepecs & Mainen, 2012). Across the three experiments, we saw some parallels between confidence and betting. Both confidence ratings (experiments 1 and 3) and betting (experiment 2) increased significantly with outcome run length. In experiment 2, the likelihood of betting high was greatest after longer outcome runs as participants committed the gambler's fallacy. This supports an assertion that betting varies in line with decision confidence, and notably, the same run length by choice interaction approached significance in experiment 1 (*p* = .07) and experiment 3 (*p* = .10). However, our clearest effect of feedback streaks was an increase in betting following losing streaks, which was not observed for subjective confidence ratings; indeed, confidence was generally higher following wins. This result indicates that while betting may scale approximately with decision confidence, it is also susceptible to selective influences from the economic context. Another example of such susceptibility to economic context was described by Fleming and Dolan (2010), who found that post-decision wagering on a stimulus-detection task showed patterns of loss aversion (refer also to Dienes & Seth, 2010). These effects contaminate the utility of betting as a measure of simple confidence, and raise a note of caution for researchers interested in using post-decision wagering.

Experiments 1 and 3 demonstrated a strong gambler's fallacy effect without any reliable effect of feedback streak length upon subjective confidence. From this, we infer that the gambler's fallacy is a more robust phenomenon in the psychological laboratory than any hot hand effect. Nevertheless, in considering our failure to replicate the seminal study by Ayton & Fischer (2004, Experiment 1), several features should be borne in mind. First, participants were generally more confident following wins than losses, but this did not vary with the length of streak. This effect, coupled with the significant effect of outcome runs on confidence in experiments 1 and 3, militate against an argument that our participants' confidence ratings were simply noisy and/or thus lacked sensitivity. It is possible that betting is a *more* sensitive measure than confidence and that experiments 1 and 3 may have therefore lacked the statistical power to detect the effect in experiment 2. If this were the case, we would expect the confidence intervals around the ORs (a measure of effect size in a logistic regression) to be wider in experiments 1 and 3 compared with experiment 2; in fact, they are narrower. However, other methodological differences may exist. A study by Matthews (2013) on outcome runs saw that the gambler's fallacy following three consecutive outcomes was sensitive to the surrounding context of shorter or longer runs. If a similar phenomenon applies to feedback streaks, then differences could emerge between studies as a result of exact sequence experienced (refer also to Ball, 2012). The overall sequence length (200 trials in Ayton and Fischer, compared with 90 in our task) may play a role (Hahn & Warren, 2009), and trait-like individual differences may exist, for example, in gambling cognitions (Marmurek et al., 2013).

#### Conclusions

The present data help to specify the experimental conditions under which the effects of outcome runs and feedback streaks are observed in the laboratory. The gambler's fallacy was consistently seen for choice behavior in all three experiments, corroborating past work on the distorted processing of random events even in well-educated student participants. Our observation that participants bet high after longer runs when choosing against the previous outcome (experiment 2) demonstrates that the gambler's fallacy also impacts on risk-taking behavior. We saw no influence of the length of feedback streaks on subjective confidence ratings (experiments 1 and 3), but betting increased as a function of losing streak length in experiment 2. This is interpreted in terms of loss chasing within a laboratory task, with two distinct implications: first, that loss chasing itself may reflect an increase in the subjective value of winning with no change in the perceived expectancy of winning, and second, that betting behavior is affected by economic context to the degree that it should not be regarded as a simple proxy for declarative confidence judgments.
